# Impact of oral mucositis treated by photobiomodulation protocol on the quality of life of pediatric oncology patients with acute lymphoblastic leukemia

**DOI:** 10.1007/s10103-026-04903-7

**Published:** 2026-06-12

**Authors:** William Alves de Melo Júnior, Gustavo Correia Basto da Silva, Fernanda Suely Barros Dantas, Raíssa Cássia Gomes Aciole, Fernando Luiz Affonso Fonseca, Maria Aparecida da Silva Pinhal

**Affiliations:** 1 Programa de Pós-Graduação em Ciências da Saúde, 1- Centro Universitário FMABC, Santo André- SP, Brazil; 2 Federal University of Campina Grande, Campina Grande, Brazil; 3 2- Depto de Odontologia Social e Preventiva, Universidade Federal de Minas Gerais, Belo Horizonte, Brazil; 4 Dep. Estomatologia, Federal University of Pernambuco, Recife, Brazil; 5 Hospital Dentistry, Hospital Universitário Alcides Carneiro - HUAC, Campina Grande, Brazil; 6 Department of Pharmaceutical Sciences, Universidade Federal de São Paulo, São Paulo, Brazil

**Keywords:** Antineoplastic therapy, Oral mucositis, Quality of life, Acute lymphoblastic leukemia, Methotrexate, Photobiomodulation therapy

## Abstract

To assess factors associated with methotrexate-induced oral mucositis and its impact on the quality of life of pediatric patients with acute lymphoblastic leukemia, considering chemotherapy dose and mucositis severity within a clinical context where photobiomodulation is routinely used as part of the institutional care protocol. A cross-sectional study was conducted with 113 pediatric patients aged 5 to 18 years undergoing methotrexate-based chemotherapy between 2020 and 2024 in a university hospital in Northeastern Brazil. Data were collected using the Children’s International Mucositis Evaluation Scale (ChIMES; self-report and proxy), the Child Oral Impacts on Daily Performances (C-OIDP), the WHO Oral Mucositis Grading Scale, photobiomodulation records, and chemotherapy regimen data. Descriptive statistics, Spearman correlation, and multiple linear regression analyses were performed (α = 0.05). Among the participants, 85% reported a high impact on quality of life. The mean age was 10.8 years (SD = 3.49), and most participants were boys. Higher mucositis severity, older age, higher methotrexate doses, and longer wound healing time were associated with worse quality of life. A strong positive correlation was observed between C-OIDP and ChIMES scores (*p* < 0.001). In the regression analysis, mucositis severity (β = 0.304; *p* = 0.021) and wound healing time (β = 0.492; *p* < 0.001) remained significantly associated with higher impact scores. Methotrexate-induced oral mucositis was associated with a substantial negative impact on quality of life in pediatric patients with acute lymphoblastic leukemia. Greater mucositis severity and longer wound healing time were the main factors associated with worse outcomes. Although photobiomodulation was systematically applied as a preventive and therapeutic intervention, no causal inference regarding its independent effect can be established.

## Introduction

Acute lymphoblastic leukemia (ALL) is the most common hematologic malignancy in childhood, accounting for the majority of pediatric cancer cases [1]. Treatment involves multiple phases of chemotherapy (CT), often including high-dose methotrexate (HD-MTX), which may lead to adverse effects such as oral mucositis (OM) and immunosuppression [2].

OM is a debilitating complication of antineoplastic treatments, potentially affecting the gastrointestinal tract and oral cavity, resulting in pain, difficulty eating, weight loss, infections, and even the need to modify or interrupt CT [3, 4]. Clinically, it begins as erythema and may progress to erosive or ulcerative lesions in the oral mucosa, tongue, floor of the mouth, and soft palate [4–6].

The severity of OM varies by cancer type, with hematologic malignancies often showing earlier onset. Risk factors include leukocyte and platelet counts, as well as the type of CT agent used [7]. Methotrexate (MTX), a key agent in the treatment of pediatric malignancies, particularly affects rapidly dividing cells in the mucosa and bone marrow, leading to myelosuppression and mucositis. HD-MTX is essential in the consolidation phase of ALL treatment [2, 7, 8].

Studies show that OM significantly impairs quality of life (QOL), extending beyond oral complications to physical, emotional, and psychological domains due to pain, poor nutritional intake, increased risk of opportunistic infections, and chemotherapy interruptions [9, 10]. To assess oral health-related quality of life, instruments such as the OHIP-14 and the Child Oral Impacts on Daily Performances (C-OIDP) have been used. The C-OIDP has demonstrated validity, reliability, and appropriate psychometric properties for use in children and adolescents [11–15].

The heterogeneity in OM impact assessment, combined with clinical examination challenges in pediatric patients, highlighted the need for a standardized approach. In this context, the Child’s International Mucositis Evaluation Scale (ChIMES) was developed, incorporating key components such as pain, swallowing, eating, drinking, and oral mucosa appearance [15]. Comprehensibility, acceptability, and validity tests have shown that the self-reported and proxy Portuguese versions of ChIMES are culturally adapted, valid, and reliable for Brazilian pediatric patients aged one month to 18 years [16, 17].

Given the significant impact of OM on QOL, especially in pediatric oncology, this study addresses the lack of data on MTX-induced OM in children with ALL. Considering the growing evidence on the benefits of photobiomodulation (PBM) in managing mucositis, the study aims to assess factors associated with OM and its impact on quality of life, exploring MTX as a predictor and highlighting PBM as a supportive therapeutic approach.

## Materials and methods

### Study design

This is a cross-sectional observational study conducted in the Pediatric Oncology Department of a university hospital in a city in Northeastern Brazil, in accordance with the Strengthening the Reporting of Observational Studies in Epidemiology (STROBE) guidelines.

### Ethical aspects

This study was conducted in accordance with the Declaration of Helsinki and was approved by the local Human Research Ethics Committee under CAAE number 40132420.6.0000.5182. Informed consent was obtained from parents or legal guardians for all participants. Assent was obtained from children aged 7 years and older, according to their level of understanding and institutional ethical guidelines.

### Participants

A convenience sample of patients aged 5 to 18 years, of both sexes, diagnosed with acute lymphoblastic leukemia and undergoing methotrexate-based chemotherapy was included. Only patients who developed oral mucositis between December 2020 and February 2024 were included. Patients with clinical conditions that prevented oral examination or data collection, those undergoing other types of antineoplastic treatments, or those presenting oral mucosal inflammation prior to chemotherapy were excluded.

### Data storage and management

All study data were de-identified prior to analysis and securely stored on password-protected institutional computers, with access restricted to the research team. Data extracted from medical records and clinical assessment forms were coded and entered into an electronic database. Source documents were linked to the analytical dataset through coded identifiers only. Data entry, management, and statistical analyses were performed using SPSS for Windows (version 25.0, IBM Corp., Armonk, NY, USA), in accordance with institutional data governance policies and ethics committee requirements.

### Data collection

Data on sex, age, diagnosis, and chemotherapy regimen were extracted from medical records. To ensure standardized assessments, two examiners were calibrated prior to data collection, resulting in high inter-examiner agreement (Kappa = 0.89). Disagreements were resolved by consensus.

During routine clinical care, oral lesions were clinically evaluated by trained dental and medical teams as part of standard hospital practice. However, no systematic laboratory investigation for opportunistic infections (such as fungal cultures, viral testing, or PCR) was performed for research purposes.

The instruments used included: (1) the Children’s International Mucositis Evaluation Scale (ChIMES), applied in self-report form (for children ≥ 7 years) and proxy form (for caregivers of children aged 5–6); (2) the Child Oral Impacts on Daily Performances (C-OIDP), using a facial analog scale for severity and a frequency chart; (3) the World Health Organization (WHO) Oral Mucositis Grading Scale; and (4) photobiomodulation session records.

The Children’s International Mucositis Evaluation Scale (ChIMES), validated for use in pediatric cancer patients, assesses mucositis through six items: four scored from 0 (best) to 5 (worst), and two dichotomous items (“yes” = 1, “no” = 0), totaling up to 22 points. The instrument was administered at bedside after chemotherapy, usually between the second and third week, when mucositis signs became evident. It was applied using both self-report and proxy versions: self-reports were obtained from children able to understand and respond to the instrument, while proxy reports were completed by caregivers for younger children, particularly those under 7 years of age.

The C-OIDP assessed the impact of mucositis on eight domains of daily life, including eating, speaking, oral hygiene, sleeping, emotional well-being, social interaction, and play. A Likert scale was used for intensity and frequency, with scores calculated by multiplying both values. The final score was standardized as a percentage (0–100%). Interviews were aided by a three-point facial analog scale representing levels of difficulty.

All patients were in the consolidation phase and underwent the Berlin-Frankfurt-Münster European Group protocol (BFM 2002), which uses methotrexate (MTX) at low or high doses (high-dose methotrexate – HD-MTX), either alone or in combination with doxorubicin (DOX) and cyclophosphamide (CTX).

All patients received a standardized baseline oral supportive care protocol as part of routine institutional practice in pediatric oncology. This protocol included supervised oral hygiene with a soft toothbrush, use of fluoridated toothpaste, lip moisturizing, pain management, nutritional support as prescribed by the medical team, and oral health education provided by the dental team. Alcohol-free 0.12% chlorhexidine mouthwash was used when clinically indicated, such as in the presence of ulcerative lesions, suspected secondary infection, or high plaque accumulation.

PBM therapy was systematically applied as part of the institutional protocol, with preventive and therapeutic purposes in the management of oral mucositis. Sessions were performed daily, starting 24 h after chemotherapy infusion, using a red laser (660 nm) in punctual mode with an energy of 1 J per point. Irradiation was applied to predefined and standardized oral sites commonly affected, with the total number of points adjusted according to oral cavity size (22–29 points). In selected cases of severe lesions associated with intense pain, an additional infrared wavelength (808 nm) was applied directly to ulcerated areas. The selection of wavelengths, treatment sites, and number of points was guided by current evidence and clinical recommendations for pediatric oral mucositis.

The procedure was performed using the Therapy EC device (DMC, São Carlos, SP, Brazil), with the following specifications: red laser wavelength of 660 nm (± 10 nm), output power of 100 mW (± 20%), spot size of 0.028 cm², energy density (fluence) of 35.7 J/cm², irradiance of 3.57 W/cm², and InGaAlP semiconductor diode.

Chemotherapy dose was recorded in milligrams (mg), as documented in medical records. Body surface area (mg/m²) was not consistently available for all patients and therefore was not used in the analysis.

### Statistical analysis

The data were subjected to descriptive and inferential statistical analyses. The descriptive analysis included absolute and relative frequencies, as well as measures of central tendency and dispersion (mean, standard deviation, and quartiles). The Chi-square and Fisher’s exact tests were applied for comparisons between the C-OIDP groups, classified according to the levels of impact on QOL. The categorization of C-OIDP scores was based on the empirical distribution of the sample. Individuals with a score of 0 were classified as “no impact,” while the remaining observations were divided into tertiles using percentile-based cutoffs to form low, medium, and high impact groups. These categories were defined for analytical purposes and do not represent clinically established thresholds.

Quantitative variables, since they did not follow a Gaussian distribution, were compared between groups using the Mann-Whitney test. Effect sizes for qualitative variables were calculated using Phi and Cramer’s V, while biserial correlation was used for numerical variables.

Spearman correlation analyses were conducted between the ChIMES score and its questions and the C-OIDP score. For categorical questions, point-biserial correlation was used. In addition, multiple linear regression analysis (enter method) was performed to investigate the impact of predictors (age, sex, mucositis grade, chemotherapy dose, wound healing time, ChIMES score) on the QOL scores measured by the C-OIDP. Statistical significance was set at *p* < 0.05.

All analyses were conducted using the Statistical Package for the Social Sciences (SPSS for Windows, version 25.0, IBM Corp., Armonk, NY, USA).

## Results

Of the 113 patients evaluated, 85% of patients were classified as having a high impact on quality of life. Most participants were boys, with a mean age of 10.8 years (SD = 3.49). Regarding chemotherapy regimen, 55.8% received MTX, 29.2% MTX-HD, and 15.0% MTX-HD combined with CTX and DOX (Table 1).

**Table 1:** Sample characteristics

Variables	*n* (%)
Total	**113 (100.0)**
Sex	
Boys	74 (65.5)
Girls	39 (34.5)
Methotrexate-based chemotherapy regimen	
MTX	63 (55.8)
MTX-HD	33 (29.2)
MTX-HD + CTX + DOX	17 (15.0)

In the bivariate analysis (Table 2), older age was associated with a greater impact on quality of life (*p* = 0.003). Higher degrees of oral mucositis were strongly associated with worse quality of life (*p* < 0.001), with a large effect size. Higher chemotherapy doses were also associated with greater impact on quality of life (*p* < 0.001). The chemotherapy dose variable showed a highly skewed distribution, as reflected by the discrepancy between mean and median values and the large standard deviation. Furthermore, longer time to mucositis resolution was significantly associated with worse quality of life outcomes (*p* < 0.001).

**Table 2:** Association between predictor variables and Quality of Life levels (C-OIDP)

Categorical independent variables	C-OIDP (medium) *n* (%)	C-OIDP (high) *n* (%)	*p*-value	Effect size
C-OIDP classification	17 (15.0)	96 (85.0)	-	-
Sex				
Boys	8 (10.8)	66 (89.2)	*p* = 0.083^a^	0.16
Girls	9 (23.1)	30 (76.9)		
Degree of mucositis( OMS)				
1	17 (94.4)	1 (5.6)	*p* < 0.001*^a^	0.967
2	0 (0.0)	40 (100.0)		
3	0 (0.0)	49 (100.0)		
4	0 (0.0)	6 (100.0)		
Quantitative independent variables	M (DP) Median (P25 - P75) Range	M (DP) Median (P25 -P75) Range	*p*-value	Effect size
Age	8.59 (2.83) 7 (6.5; 10.5) 5–15	11.19 (3.47) 11 (8; 14.0) 5–18	*P* = 0.003^*b^	0.28
Chemotherapy dose**	21.82 (9.89) 18 (15.5; 26) 12–42	919.17 (1572.36) 155 (80; 1088.0)	*p* < 0.001^*b^	0.60
Wound healing time (days) ***	5.12 (0.93) 5 (4.5; 5.5) 4–7	8.56 (2.49) 8 (7; 10.0) 5–14	*p* < 0.001^*b^	0.52

*C-OIDP* child oral impacts on daily performances, *n* absolute frequency; *M* mean, *P* percentile; *p < 0.05 indicates statistical significance; ** in milligrams; *** in days; a Pearson’s chi-square test; b Mann–Whitney test

Table 3 presents the Spearman correlation between ChIMES domains and the C-OIDP total score. All domains showed significant positive correlations (*p* < 0.001), indicating that greater severity of oral mucositis symptoms was associated with a higher impact on oral health–related quality of life. Strong correlations were observed for pain in the mouth or throat, difficulty eating, difficulty swallowing saliva, and difficulty drinking, while moderate correlations were found for use of pain medication, use of medication because of pain in the mouth or throat, and appearance.

**Table 3:** Correlation between ChIMES domains and C-OIDP total score (Spearman’s correlation)

ChIMES domains	ρ (rho)	*p*-value
Pain in the mouth or throat	0.944	*p* < 0.001
Difficulty swallowing saliva	0.935	*p* < 0.001
Difficulty eating	0.948	*p* < 0.001
Difficulty drinking	0.942	*p* < 0.001
Use of pain medication	0.666	*p* < 0.001
Use of medication due to pain in the mouth or throat	0.848	*p* < 0.001
Appearance	0.664	*p* < 0.001

Spearman’s correlation coefficient (ρ) was used. All correlations were statistically significant (*p* < 0.001)

The regression model showed a significant overall effect on C-OIDP scores (F(6, 106) = 65.439, *p* < 0.001; adjusted R² = 0.775). Table 4 presents the regression coefficients for all predictors. Wound healing time had the strongest impact on C-OIDP scores (β = 0.492, *p* < 0.001), followed by the degree of mucositis (β = 0.304, *p* = 0.021).

**Table 4:** Multiple linear regression analysis for C-OIDP score showing unstandardized and standardized coefficients

Variables	C-OIDP score			
	b	Beta	t	*p*-value
(Constant)	-10.381	-	-1.923	0.057
Age	-0.280	-0.052	-1.119	0.265
Sex	0.720	0.018	0.403	0.688
Degree of mucositis	6.972	0.304	2.352	0.021*
Chemotherapy dose	0.000	0.027	0.475	0.636
Wound healing time (days)	3.510	0.492	6.489	< 0.001*
ChIMES score	0.427	0.145	1.196	0.234

*C-OIDP* oral impacts on daily performances; b: Unstandardized regression coefficient; Beta: Standardized regression coefficient; * *p* < 0.05

## Discussion

This study investigated factors associated with oral mucositis severity and its impact on oral health–related quality of life in pediatric patients with acute lymphoblastic leukemia receiving chemotherapy within a standardized supportive care protocol that included PBM.

The findings demonstrated that mucositis severity and wound healing time were significantly associated with higher C-OIDP scores, indicating a substantial impact on daily functioning.

Regarding the chemotherapy regimen, methotrexate (MTX) was adopted as an eligibility criterion and administered during the consolidation phase, either alone or in combination with cyclophosphamide (CTX) and doxorubicin (DOX) in intermediate- and high-risk groups. MTX is a key chemotherapeutic agent widely used in the treatment of hematological malignancies, particularly acute lymphoblastic leukemia (ALL), the most common childhood cancer [1, 2, 9].

Despite its therapeutic relevance, MTX is associated with a range of adverse effects, including toxicities affecting the skin, oral mucosa, liver, and kidneys, which may limit its clinical use [18].

In the present study, higher grades of oral mucositis (grades 3 and 4) were more frequently observed in patients receiving high-dose MTX, particularly when combined with CTX and DOX. These findings are consistent with previous studies, such as Curra et al. [5], and with evidence indicating that high-dose MTX regimens are associated with increased incidence and severity of oral mucositis [10, 18, 19].

Although chemotherapy dose was not retained as a significant predictor in the adjusted regression model, its distribution was highly variable, and descriptive analyses suggested a trend toward more severe mucositis in patients exposed to higher doses. Previous studies have demonstrated that high-dose MTX is associated with an increased risk of oral mucositis, with reported incidence ranging from 20% to 50% [18]. However, mucositis may also occur at lower doses, affecting approximately 11% to 17% of patients [10, 20, 21].

In our sample, most patients (85%) presented a high impact on quality of life. Although a higher proportion of boys was observed, the role of sex as a risk factor for oral mucositis remains inconclusive. While some studies have identified female sex and older age as potential risk factors [3, 22], the present findings did not confirm this association.

Oral mucositis is a debilitating condition that can impair nutritional intake, increase the need for analgesics, prolong hospitalization, and elevate treatment costs, ultimately compromising patients’ quality of life [8]. A systematic review by Al-Rudayni et al. [11] highlighted that the impact of oral mucositis extends beyond local manifestations, affecting physical, emotional, and functional domains.

In the present study, most patients presented moderate to severe oral mucositis (grades 2 and 3), which was associated with greater impact on quality of life. These findings reinforce the clinical relevance of mucositis severity as a determinant of functional impairment, particularly in domains such as eating, speaking, swallowing, and oral hygiene.

Consistent with these observations, oral mucositis severity is associated with greater impairment in quality of life, in agreement with previous studies [3, 5, 11, 12, 23]. Furthermore, the strong correlation between ChIMES and C-OIDP scores suggests that increased symptom severity is closely related to greater impact on patients’ daily functioning.

Several instruments have been proposed for the assessment of oral mucositis; however, few are specifically designed for pediatric populations. In this study, the ChIMES scale was used, as it incorporates patient-reported symptoms such as pain in the mouth or throat, along with functional limitations related to swallowing, eating, and drinking, supporting its validity and applicability in this population [24]. In addition, the C-OIDP was used as a specific instrument to assess the impact of oral conditions on the quality of life of children and adolescents [14, 16, 24].

The analysis of ChIMES domains revealed that pain in the mouth or throat, difficulty eating, and difficulty swallowing saliva were the most impactful factors on quality of life. These findings suggest that mucositis-related symptoms are not only individually relevant but also interact to exacerbate functional limitations and negatively affect daily activities. Similar observations have been reported by Al-Rudayni et al. [11], who highlighted the multidimensional impact of mucositis on physical and social well-being.

Consistent with previous literature, oral mucositis was associated with reduced quality of life, potentially leading to delays in chemotherapy administration and prolonged disease course [25–27].

Among the predictor variables analyzed, wound healing time and mucositis severity showed the strongest associations with C-OIDP scores. Wound healing time, in particular, demonstrated the greatest impact, indicating that longer healing periods are associated with greater impairment in quality of life.

Prolonged mucositis may increase exposure to adverse clinical conditions, such as odynophagia and dysphagia, which can compromise nutritional status, increase the risk of infection, and potentially interfere with the continuity of antineoplastic treatment [10].

In most cases, OM heals spontaneously within approximately 21 days after infusion of the medication [27]. This process results from complex biological processes, where the extracellular matrix (ECM) of the submucosa guides the proliferation, migration, and differentiation of the epithelium that borders the ulcer [6].

In this sense, wound healing time, defined as the time to mucositis resolution, is an important factor in understanding the magnitude of oral mucositis severity and its impact on quality of life. PBM has been suggested to modulate inflammatory responses and enhance cellular activity, potentially contributing to tissue repair and lesion resolution [28, 29]. However, in the present study, no causal inference regarding its effect can be established, as PBM was systematically applied as part of the institutional supportive care protocol. Therefore, findings related to lesion duration should be interpreted within this clinical context, without attributing an independent therapeutic effect to PBM.

Although evidence on this topic remains limited in pediatric oncology, some studies have explored lesion duration in patients receiving PBM. Most available data are derived from adult populations and focus on comparisons between PBM alone and its association with photodynamic therapy (PDT). Pinheiro et al. (2019) [30] reported an average of 17 days for wound healing with PBM alone, while the association with PDT reduced this time to 12 days. Similarly, Lessa et al. (2023) [31] observed a mean healing time of 18.37 days (± 12.12) in patients treated with PBM + PDT, compared to 23 days (± 14.78) in those receiving PBM alone.

In pediatric populations, evidence remains scarce. In a case-control study, Melo Junior et al. (2016) [32] observed a statistically significant reduction in healing time, with the control group presenting a mean of 4.30 (± 2.8) days compared to 3.83 (± 2.7) days in the PBM group. In another similar study, most cases showed lesion resolution between 4 and 7 days [28]. These findings are consistent with the present results, in which the mean wound healing time was 6.2 (± 1.3) for mild mucositis (grades 1 and 2) and 10 (± 2.1) for severe mucositis (grades 3 and 4).

This study is strengthened by its integrated methodological approach, combining different complementary instruments to assess oral mucositis and its impacts in pediatric oncology patients with ALL undergoing CT with MTX.

The combined use of ChIMES, in its self-report and proxy versions, and C-OIDP, with facial analog scale and frequency chart, allowed a multidimensional approach, which encompasses both the child’s perception and the functional effects on QOL. In addition, the inclusion of the OM Grading Scale and the monitoring of PBM sessions provide greater clarity on the impact of OM on QOL and clinical robustness to the study, by associating subjective and objective data in a homogeneous and highly vulnerable sample.

This study has several limitations that should be considered. The absence of a control group and the inclusion of PBM as part of routine institutional care for all participants prevent causal inference and preclude comparative evaluation of its effectiveness. These findings should be interpreted within the clinical context in which PBM was systematically applied as a preventive and therapeutic intervention.

The use of chemotherapy dose recorded in milligrams (mg), without adjustment for body surface area (mg/m²), limits comparability with other studies and may affect dose–response interpretation. Furthermore, only patients who developed oral mucositis were included, which precludes the assessment of incidence and limits the identification of true risk factors. In addition, important clinical variables, such as hematological parameters, methotrexate serum levels, and nutritional status, were not included in the analysis, which may have influenced the observed associations and should be considered in the interpretation of the results.

Moderate multicollinearity between mucositis severity and ChIMES scores was identified, as these variables reflect overlapping clinical and patient-reported constructs. Although both were retained due to their clinical relevance, this may have influenced the stability and precision of regression estimates and contributed to an inflation of the model’s explanatory power.

In addition, no systematic microbiological investigation of opportunistic infections was performed. As oral lesions were clinically assessed during routine care, a potential overlap between oral mucositis and infectious conditions cannot be ruled out, which may have influenced symptom severity, pain perception, and quality of life outcomes. The absence of photographic documentation may also limit the objectivity of clinical assessments. Furthermore, the reliance on proxy reporting for younger children may have introduced measurement bias.

Finally, the single-center design and the use of a convenience sample limit the generalizability of the findings.

Future studies should adopt longitudinal and controlled designs, preferably multicenter, to better elucidate the relationship between oral mucositis severity, treatment variables, and quality of life outcomes, as well as to more robustly evaluate supportive care strategies in pediatric oncology.

## Conclusion

MTX-induced oral mucositis (OM) was associated with a substantial impact on the quality of life (QoL) of children and adolescents with acute lymphoblastic leukemia (ALL), particularly in cases of greater clinical severity. The observed correlations between ChIMES and C-OIDP scores support the relationship between clinical manifestations and patient-reported outcomes. Chemotherapy dose was associated with increased mucositis severity, while wound healing time and severity emerged as key factors related to QoL impairment.

These findings underscore the relevance of strategies aimed at preventing and controlling OM within the context of intensive chemotherapy protocols. Although all participants received photobiomodulation (PBM) as part of institutional supportive care, no causal inference regarding its effectiveness can be established in the present study.

Future multicenter studies with larger samples are warranted to further investigate the impact of OM on QoL and to strengthen evidence-based supportive care approaches in pediatric oncology.

## Data Availability

The datasets generated and/or analyzed during the current study are not publicly available due to ethical and privacy restrictions involving pediatric patients, but are available from the corresponding author on reasonable request and subject to institutional and ethical approval.
